# Repositioning Quinacrine Toward Treatment of Ovarian Cancer by Rational Combination With TRAIL

**DOI:** 10.3389/fonc.2020.01118

**Published:** 2020-07-16

**Authors:** Rui Liang, Yuanfei Yao, Guangyu Wang, Er Yue, Guangchao Yang, Xiuying Qi, Yang Wang, Ling Zhao, Tongsen Zheng, Yanqiao Zhang, Edward Wenge Wang

**Affiliations:** ^1^Department of Medical Oncology & Therapeutics Research, City of Hope Comprehensive Cancer Center and Beckman Research Institute, Duarte, CA, United States; ^2^Department of Pharmacy, Suzhou Vocational Health College, Suzhou, China; ^3^Cancer Hospital, Harbin Medical University, Harbin, China

**Keywords:** quinacrine, TRAIL (TNF-related apoptosis-inducing Ligand/Apo2L), DR5, ovarian cancer, lysosomal permeabilization, intraperitoneal (IP), xenograft animal model

## Abstract

Quinacrine has been identified as a potent DR5-inducing agent that sensitizes cancer cells to TRAIL-induced apoptosis. In the current study, we found that quinacrine increased DR5 mRNA levels significantly in ovarian cancer cell lines regardless of p53 status. Further study showed the half-life of DR5 in quinacrine-treated cells was significantly prolonged, indicating that DR5 protein degradation was inhibited by quinacrine. We tested if the combination of TRAIL and quinacrine could be effective in ovarian cancer treatment *in vitro* and in ovarian cancer xenograft mouse models. We found that quinacrine enhanced TRAIL sensitivity or reversed TRAIL resistance in all the ovarian cancer cell lines tested. Mice treated with quinacrine and TRAIL remained disease-free for up to 20 weeks, however, mice treated with TRAIL or quinacrine alone and in control group died within ~8 weeks after treatment. Intraperitoneal delivery of quinacrine and TRAIL is rational and practical with extraordinary synergistic anti-cancer effects in preclinical models of ovarian cancer. Clinical investigation of combining quinacrine with TRAIL for ovarian cancer treatment is warranted.

## Introduction

The extrinsic apoptotic pathway has been well-investigated and characterized; however, targeting this pathway for cancer treatment has not been successful (1, 2). The death receptor ligand TRAIL (tumor necrosis factor-related apoptosis-inducing ligand) appears to be an ideal cancer therapeutic with minimal toxicity in preclinical models. It can induce cell death in cancer cells but not normal cells (3–5). Phase I studies of recombinant human TRAIL showed it was well-tolerated with a half-life range from 0.56 to 1.02 h (6). However, further clinical studies on various human cancers in combination with different standard chemotherapy regimens showed no remarkable synergistic anti-cancer effects (7–9), and in a trial for treatment of non-small cell lung cancer, TRAIL plus chemotherapy only moderately prolonged progression free survival (PFS) (10). Due to limited clinical benefit, clinical trials using recombinant human TRAIL, as well as agonist antibodies to DR4 and DR5 (death receptors) were suspended.

TRAIL induces apoptosis in cancer cells by engaging death receptors, DR4 and DR5. Subsequently pro-caspase 8 is cleaved to become active caspase 8 by recruiting FADD (fas-associated death domain) to form DISC (death-inducing signaling complex). Cleaved caspase 8 then induces a series of caspase reactions and eventually activates caspase 3/7 to execute extrinsic apoptosis pathway. In most circumstances, cleaved caspase 8 also activates bid, which sends death signals to mitochondrion and simultaneously promotes intrinsic pathways to induce apoptosis. Death signaling in normal cells is well-controlled and regulated, either by limiting production of death receptors or facilitating degradation to keep a low level of death receptors. It has been reported that negative regulators such as FLIP (FLICE [Fas-associated death-domain-like IL-1beta-converting enzyme]-inhibitory proteins) could prevent DISC activation (11). Subcellular localization of death receptors may also restrict access of TRAIL to its receptors and result in TRAIL resistance (12). The discrepancy between preclinical and clinical outcomes with respect to TRAIL may be explained by TRAIL resistance in primary cancer cells from patients (1) that have a higher threshold for reactivation of the extrinsic pathway than in preclinical models. TRAIL and other agonist antibodies against death receptors are not potent enough to elicit a significant anti-cancer effect in clinical studies. Therefore, overcoming TRAIL resistance in cancer cells is critical to successfully targeting the extrinsic pathway for cancer treatment.

Despite attempts to reverse TRAIL resistance using DNA-damaging agents (13), kinase inhibitors (14), immune checkpoint inhibitors (15), and other targeting agents, none have shown clinical significance. While searching for small molecules targeting p53 (16–18), we identified a category of small molecules including quinacrine that induced substantial high levels of DR5 and enhanced TRAIL sensitivity or reversed TRAIL resistance in almost all cancer cell lines tested (16, 17, 19). Further studies revealed the underlying mechanism by which these small molecules block the lysosomal degradation pathway and result in accumulation of death receptors and the associated DISC in the cytoplasm, sustaining death signaling and reversing TRAIL resistance. Quinacrine, a bioactive acridine derivative that has been used as an anti-malarial agent for treatment and prophylaxis, stood out as a potent extrinsic pathway activator able to reverse TRAIL resistance in human cancer cells, including ovarian cancer cell lines. These studies provided the rationale to test the combination of quinacrine and TRAIL for ovarian cancer treatment.

In this work, we demonstrated that quinacrine induced high level of DR5 and reversed TRAIL resistance in human ovarian cancer cell lines. Quinacrine not only significantly induces high mRNA level of DR5, but also reducesDR5 protein degradation, most likely in lysosomes. Further experiments showed that quinacrine induces permeabilization of lysosomal membranes, as manifested by aggregation of galectin-3 in lysosome, which may also contribute to the synergistic effect of quinacrine and TRAIL in executing cancer cell death. Surprisingly, interperitoneally delivery of TRAIL and quinacrine showed remarkable synergistic effect in treating mice bearing A2780 ovarian cancer xenografts. Our data provides conclusive evidence for rational combination of quinacrine and TRAIL for ovarian cancer treatment.

## Materials and Methods

### Cell Culture and Chemicals

Ovarian cell lines SK-OV-3, OVCAR-4, OVCAR-8, and A2780 were cultured in RPMI-1640 (Gibco) containing 10% Fetal Bovine Serum (Neuromics, Edina, MN), L-glutamine and 1% penicillin-streptomycin in a humidified incubator (5% CO_2_) at 37°C. Quinacrine, z-VAD-fmk, and cycloheximide (CHX) were obtained from Millipore Sigma (St. Louis, MO). D-Luciferin, potassium salt, was purchase from BioVision (Milpitas, CA).

### Cell Viability Assay

The Cell Counting Kit-8 (CCK-8) for cell viability assay was purchased from Dojindo Molecular Technologies (Rockville, MD). Cells were plated in 96-well plates in culture overnight and treated with TRAIL, quinacrine, or in combination at indicated concentrations for 16 h. CCK-8 solution was added to each well; 10 μl/well. Absorbance at 450 nm was measured by the Tecan Spark 10M multimode microplate reader. Drug synergy analysis was conducted using CompuSyn software (20). Alternatively, we used Coomassie blue stain to visualize live cells attached to culturing plate. OVCAR-8 and A2780 cells growing nearly 100% confluency in 12-well plates were treated with TRAIL 100 ng/ml, quinacrine 20 μM, or in combination for 16 h. Then died or floating cells were washed out with PBS for 3 times. Attached live cells were stained with 0.1% Coomassie Brilliant Blue R-250 (Research Products International, Mt Prospect, IL) in 50% methanol and 10% glacial acetic acid for 10 min. The plate wells were washes 3 times with tab water and dried for image scan.

### Caspase Assay

The Caspase-Glo® 3/7 Assay kit was purchased from Promega (Madison, WI). Ovarian cancer cells were plated in a black 96-well plate with clear bottom. After overnight incubation, cells were treated for 16 h with TRAIL, quinacrine, pan-caspase inhibitor z-VAD-fmk or in combination. Luminescence intensity was recorded by a Tecan Spark 10M multimode microplate reader and caspase 3/7 activity was calculated per the manufacturer's instructions.

### Plasmid Construction, Recombinant Protein Purification, Retrovirus, and Lentivirus Production

The human TRAIL cDNA fragment (coding amino acids 94–281) was codon optimized, synthesized by IDT (Integrated DNA Technologies, Inc. Coralville, Iowa) and inserted into a bacterial expression vector, pQE80L (Qiagen) to generate a His-tagged human recombinant TRAIL, which was purified per a protocol published previously (21). The human DR5 cDNA fragment (coding amino acids from 1 to 334) was codon optimized and synthesized by IDT, fused with EGFP at the C-terminus, and cloned into the pBabe-puro retroviral vector. The ORF of galectin-3 was codon optimized and synthesized by IDT and cloned into thepLVEF-1a lentivirus expression vector (kindly provided by Dr. Guihua Sun, Diabetes & Metabolism Research Institute at City of Hope) fused with mCherry at the N-terminus. For retrovirus production, the pBabe/DR5-EGFP plasmid was transfected into Phoenix cells for packaging. Virus containing supernatant was harvested 48–72 h post-transfection for target cell infection in the presence of polybrene at 10 μg/ml. The lentivirus vector pLVEF-1a-mCherry-galection-3 was co-transfected into 293T cells with packaging plasmids for lentivirus production. Virus containing supernatant was harvested 48–72 h post-transfection and purified for target cell infection in the presence of polybrene at 10 μg/ml.

### Antibodies and Western Blotting

Antibodies against human caspase-3, caspase-8, caspase-9, and DR5 were purchased from Cell Signaling Technology (Danvers, MA). Antibodies against p53, DO1, was purchased from Santa Cruz Biotechnology (Dallas, TX). The Ran antibody was purchased from BD Biosciences (Franklin Lakes, NJ). Cell lysates were prepared using RIPA lysis buffer. Protein samples were subjected to SDS-PAGE on 4–20% mini-protein TGX gels (Bio-Rad, CA, USA) and transferred onto Immobilon™ membranes (Millipore, MA). The membrane was blocked using blocking buffer for 1 h and probed with primary antibody for 1 h at room temperature or overnight at 4°C, washed, and then subjected to secondary antibodies labeled with IRDye (LI-COR Biosciences; Lincoln, NE). Blots were scanned using the Odyssey CLx Imaging System (LI-COR).

### Quantitative Real Time PCR (qPCR)

Total RNA was extracted from cells using the RNeasy Mini Kit (Qiagen, Germantown, MD). Complementary DNA was synthesized using the Super-script III first-strand cDNA synthesis Kit (Invitrogen, Carlsbad, CA). For quantitative real-time PCR, cDNA and specific primer pairs were mixed with Power SYBR Green PCR Master Mix (Applied Biosystems, Warrington, UK), and run on the ABI Prism 7900HT Sequence Detection System (Applied Biosystems, Warrington, UK). The following primers were used for amplification of DR5: DR5 (sense) 5′-ATGGAACAACGGGGACAGAAC-3′ and (antisense) 5′-CTGCTGGGGAGCTAGGTCT-3′. The threshold cycle number (Ct) for gene expression was calculated, and GAPDH was used as a reference. Delta-delta Ct values for gene expression were presented as relative fold induction.

### Flow Cytometry

Cells were treated with the test compounds for 16 h and cells were collected upon trypsinization and centrifugation. The pellets were washed with PBS and incubated with Annexin V and DAPI (BD Biosciences, San Jose, CA) in binding buffer for 15 min. Then, 400 μl of cells in binding buffer was loaded and cells were analyzed by flow cytometry.

### Immunofluorescent Staining

A2780 cells in a chamber slide treated with quinacrine (15 μM, 4 h) were fixed with 4% formaldehyde solution in PBS buffer (pH 7.4) for 10 min in room temperature, then blocked in 3% bovine serum albumin in PBS for 30 min. Anti-Lamp2 (Cell Signaling Technology, Danvers, MA) was added and incubated for 1 h. Goat anti-rabbit IgG AlexaFluor-568 (Invitrogen, Carlsbad, CA) was used as secondary antibody. Nucleus was counterstained with DAPI (Vector Laboratories Burlingame, CA). Images were captured with a ZEISS LSM 880 confocal laser scanning microscope (White Plains, NY).

### Tumor Xenograft Studies

All animal experiments were carried out in accordance with animal care guidelines approved by the Institution Animal Care and Use Committee (IACUC) of City of Hope. Female NSG mice (6–8 weeks old with body weight range from 19 to 23 grams) were purchased from The Jackson Laboratory. The animals were acclimated for 1 week prior to the study. A2780-luc cells (5 × 10^6^) in 100 μL of PBS were injected intraperitoneally into NSG mice. Five days later tumor cells in the mice were imaged after intraperitoneal injection of 2 mg/mouse D-luciferin by SPECTRAL Lago X Imaging System (Spectral Instruments Imaging, Tucson, AZ). Bioluminescent images were captured and the intensity was quantified using the Aura Imaging Software developed by Spectral Instruments Imaging as a baseline on day 0. Mice were then randomly divided into four groups and treated with quinacrine, TRAIL, quinacrine plus TRAIL, or control with vehicle as follows: quinacrine was delivered at a dose of 50 mg/kg on day 1. TRAIL was injected intraperitoneally at 10 mg/kg on day 1, 2, 3, and 4. On day 5, mice were reimaged using the Lago X Imaging System. A repeated cycle was started on day 7. Mice were monitored closely and euthanized when cancer-related symptoms developed such as weight loss, dehydration, abdomen distention, and impaired ambulation per the IACUC protocol.

### Statistical Analysis

For data analysis, experimental samples were compared to control by one-way variance analysis (ANOVA) and two-way variance analysis. Differences between groups were considered statistically significant when the *p* < 0.05. All statistical analyses were performed using the GraphPad Prism program (GraphPad Software Inc.). Combination index was calculated using CompuSyn software (22) developed by ComboSyn, Inc. Synergy, additivity, and antagonism are defined as CI < 1, CI = 1, and CI > 1, respectively.

## Results

### Quinacrine Synergizes With TRAIL in Inducing Apoptosis in Ovarian Cancer Cells

**Figure 1:**
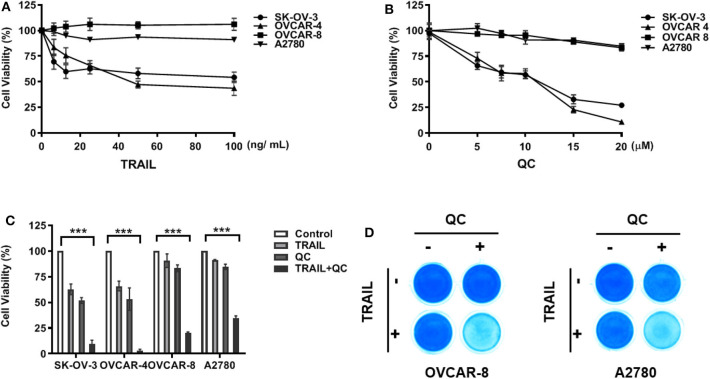
*In vitro* synergistic anti-cancer effect of quinacrine in combination with TRAIL against human ovarian cancer cells. Ovarian cancer cell lines were treated with TRAIL **(A)** or quinacrine (QC) **(B)** with the indicated dose for 16 h and subjected to the CCK-8 assay. **(C)** SK-OV-3 and OVCAR-4 were treated with 25 ng/ml TRAIL, 10 μM quinacrine, or in combination for 16 h; OVCAR-8 and A2780 cell lines were treated with 100 ng/ml TRAIL, 20 μM quinacrine, or in combination for 16 h. **(D)** OVCAR-8 and A2780 cells in 12-well plates were treated with TRAIL 100 ng/ml, quinacrine 20 μM, or in combination for 16 h, then subjected Coomassie blue stain. ***p < 0.001. All experiments were performed at least three times, reproducible.

We chose four commonly used ovarian cancer cell lines and tested their sensitivity to TRAIL—A2780, an ovarian adenocarcinoma, endometrioid type cell line; OVCAR-4, a grade 2 ovarian adenocarcinoma cell line; OVCAR-8, a grade 3 ovarian adenocarcinoma cell line; and SK-OV-3, a high grade serous ovarian carcinoma cell line. OVCAR-4 and SK-OV-3 were relatively sensitive to TRAIL, while A2780 and OVCAR-8 were resistant to TRAIL (Figure 1). After treatment with TRAIL alone at a concentration of 25 ng/ml for 16 h, less than half of SK-OV-3 cells and OVCAR-4 cells died, while following treatment together with quinacrine, almost all the cells died over 16 h, indicating a significant synergistic effect of quinacrine and TRAIL with a combination index (CI) below 1 (Supplementary Figure 1). The synergistic effect of the combination is more robust in TRAIL-resistant OVCAR-8 and A2780 cells (Figures 1C,D and Supplementary Figure 1).

**Figure 2:**
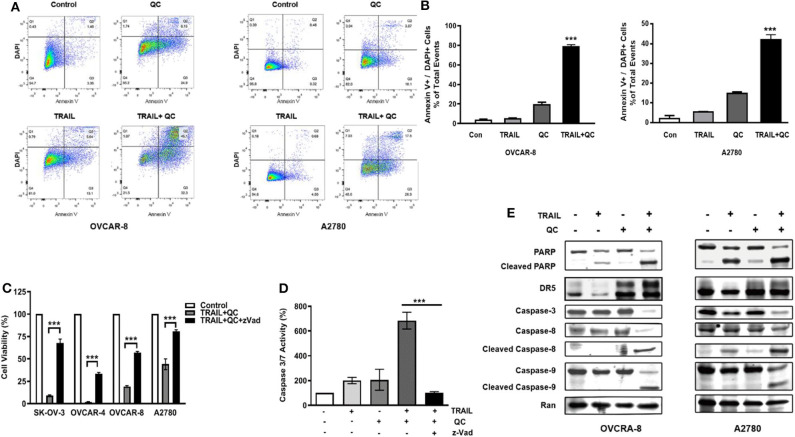
Apoptosis induced by quinacrine and TRAIL in ovarian cancer cells. **(A)** Flow cytometry analysis by Annexin V/DAPI dual staining on cells treated with quinacrine (QC), TRAIL, or combination. **(B)** Apoptotic cells positive for Annexin V and DAPI, ***p < 0.001. **(C)** SK-OV-3 and OVCAR-4 were treated with 25 ng/ml TRAIL and 10 μM quinacrine, or in combination with caspase inhibitor zVad (20 μM); OVCAR-8 and A2780 were treated with 100 ng/ml TRAIL and 20 μM quinacrine, or in combination with zVad (20 μM). Cell viability tested by CCK-8 assay. **(D)** OVCAR-8 was treated with 100 ng/ml TRAIL and 20 μM quinacrine alone or in combination, and with the caspase inhibitor zVad (20 μM). Caspase 3/7 activity was tested 12 h after treatment by Promega caspase-glo 3/7 assay. ***p < 0.001. **(E)** Western blot of PARP, DR5, caspase-8 and caspase-9, caspase-3, in OVCAR-8 and A2780 cells treated with 100 ng/ml TRAIL, 20 μM quinacrine, or combination for 16 h.

We further confirmed that quinacrine induces apoptosis in combination with TRAIL by flow cytometry using Annexin V and DAPI stain (Figure 2). Apoptosis is defined as positivity of Annexin V and DAPI stain (Figure 2B). Quinacrine or TRAIL alone failed to induce significant apoptotic cells, while combination of quinacrine and TRAIL induced significant apoptosis in cells double positive for Annexin V and DAPI stain (Figures 2A,B).

We measured caspase 3/7 activity in quinacrine and TRAIL treated cells. Combination of TRAIL and quinacrine induced significant caspase 3/7 activity, while quinacrine or TRAIL alone failed to induce significant caspase 3/7 activity at the concentration and the time point illustrated (Figure 2D). Pre-treatment with z-Vad-fmk (zVad), a pan-caspase inhibitor (23), significantly rescued TRAIL/quinacrine-induced cell death in all four cell lines (Figure 2C). Caspase 3/7 activity induced by combination of quinacrine and TRAIL was also effectively blocked by zVad (Figure 2D). Cleavage of both caspase 8, a key caspase in the extrinsic apoptotic pathway, and caspase 9, a key caspase in the intrinsic pathway, indicated both apoptotic pathways were activated by combination of quinacrine and TRAIL (Figure 2E). As a consequence, caspase 3 in the common pathway was also cleaved, as well as PARP1 (Figure 2E), indicating an irreversible apoptosis process induced by TRAIL and quinacrine.

### Quinacrine Induces DR5 mRNA Levels and Blocks DR5 Protein Degradation

**Figure 3:**
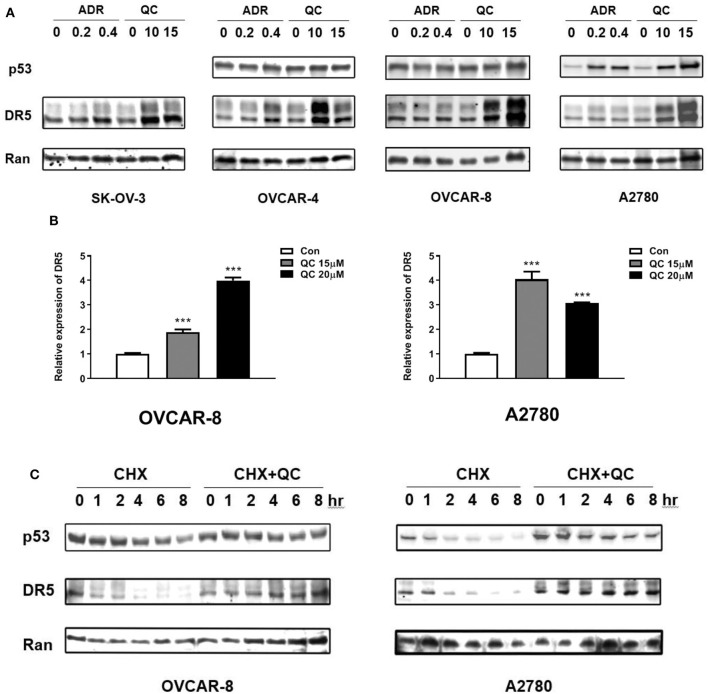
Quinacrine up-regulates DR5 through increasing of DR5 transcription and blocking DR5 protein degradation. **(A)** Ovarian cancer cell lines were treated with the indicated dose of Adriamycin (ADR) (μg/ml) or quinacrine (μM) for 8 h; p53, DR5, and Ran (loading control) were detected by Western blotting. p53 was not detected in p53-null SK-OV-3 cells (data not shown). **(B)** OVCAR-8 and A2780 were treated with quinacrine for 8 h. The mRNA level of DR5 was determined by real-time PCR. GAPDH was used as an internal control. ***p < 0.01 compared to the control. **(C)** OVCAR-8 and A2780 cells were treated with 20 μM quinacrine for 2 h, and then cycloheximide (CHX) was added at 50 μg/ml. Cells were collected at the indicated time points for Western blotting.

We tested TRAIL receptor DR5 protein and mRNA levels. As shown in Figures 2E, 3A, treatment with quinacrine induced significant protein levels of DR5 in the four ovarian cancer cell lines tested. We also tested p53 level in these cell lines treated with quinacrine because it is well-known that DR5 is a transcriptional target of p53 and quinacrine can stabilize p53 in a manner that is different from DNA-damage (16, 17). We used Adriamycin, a DNA-damage agent, as a positive control for p53 activation. Treatment of quinacrine or Adriamycin increased p53 protein levels equivalently in A2780 cells that carry a wild-type p53, while DR5 levels were significantly higher in quinacrine-treated A2780 cells compared to Adriamycin-treated cells (Figure 3A). No p53 was detected by Western blot in SKOV3 cell line, which is known to be a p53-deficient (24). The levels of p53 in OVCAR-4 and OVCAR-8 cells, both carry a mutant p53, were not changed after treatment of quinacrine or Adriamycin.

We further investigated whether the elevated DR5 protein level is due to increased production or reduced degradation. We treated OVCAR-8 and A2780 cells with quinacrine at indicated concentrations for 8 h. DR5 mRNA levels were significantly induced in OVCAR-8 and A2780 cells by quinacrine (Figure 3B). In the meantime, we tested the half-life of DR5 protein in these cells. Once protein synthesis was blocked by cycloheximide (CHX), DR5 protein decreased rapidly within 1–2 h. However, quinacrine stabilized DR5 protein level without significant decrease in up to 8 h tested (Figure 3C). These data indicate that quinacrine is a unique agent that can not only increase DR5 production by activating mRNA transcription but also block protein degradation.

### Quinacrine Induces Subcellular Localization Change of DR5 and Accumulation in Lysosomes

**Figure 4:**
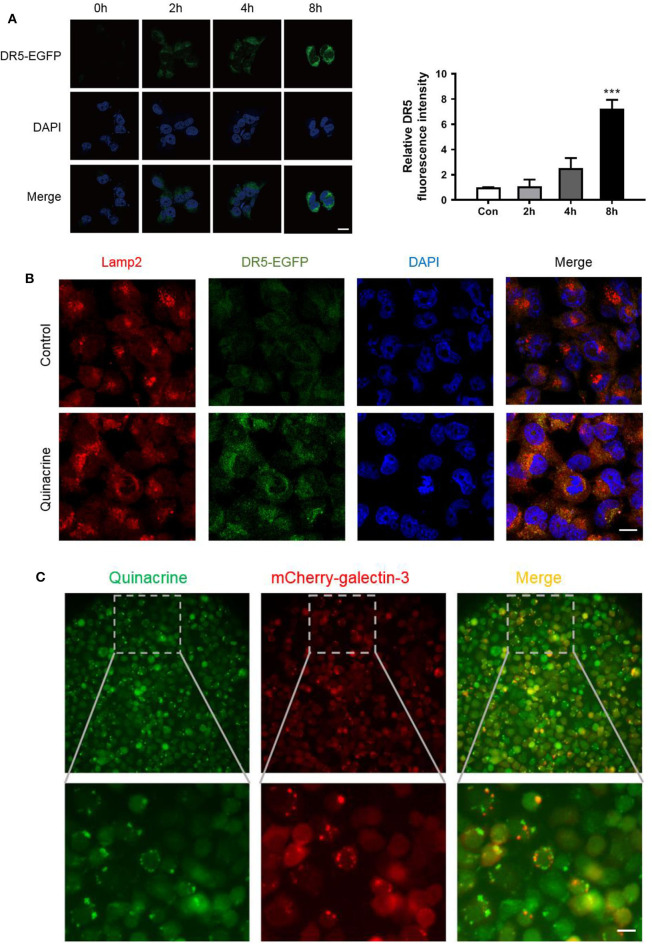
Quinacrine induces LMP and DR5 accumulation in lysosome. **(A)** OVCAR-8 expressed with DR5-EGFP was treated with 15 μM quinacrine for 2, 4, 8 h. Left, confocal images. Scan bar, 20 μm. Right, quantified fluorescence intensity. Quantification of the fluorescent intensity was performed using Image J. ***p < 0.001. All experiments were performed at least three times, reproducible. **(B)** Colocalization of DR5 and Lamp-2 was observed after treatment with 15 μM quinacrine for 4 h in A2780 cells. Scan bar, 20 μm. **(C)** Quinacrine induces LMP after 4 h as indicated by aggregated galectin-3 puncta (red, middle) in A2780. Quinacrine is a fluoresced chemical visualized in green under a laser microscope (left panel). Colocalization of quinacrine and galectin-3 puncta in lysosome (right panel). The image below is an enlargement of framed image above. Scan bar, 20 μm.

To track DR5 in the cells, we constructed a retroviral vector, pBabe/Puro/DR5-EGFP, expressing a fragment from amino acid 1–334 with the death domain deleted, fused with EGFP, and codon optimized for better expression in mammalian cells. Cancer cells infected with the retrovirus carrying DR5-EGFP and selected by puromycin showed weak expression of DR5 on the cell surface with rare intracellular deposits visualized under a laser microscope. After treatment with quinacrine for as little as 1 h, intracellular deposits of DR5-EGFP manifested as bright dots or vesicular structures increased significantly (Figure 4A). We counterstained the cells with an antibody against lysosomal membrane marker LAMP-2 (lysosomal-associated membrane protein 2) and confirmed quinacrine-induced DR5 localization in lysosomes (Figure 4B).

As fluoresce of EGFP is usually quenched in an acidic environment such as in lysosomes, the bright green signal of DR5-EGFP accumulation indicates elevated pH in lysosomes. One of the major causes of pH elevation in lysosome is lysosomal membrane permeabilization (LMP). To assess whether quinacrine treatment led to LMP, we constructed a lentivirus vector expressing galectin-3 fused with mCherry at the N-terminus, codon optimized for optimized expression in mammalian cells, and overexpressed in A2780 cells. We treated the cells with quinacrine and then visualized the expression pattern of galectin-3, which is normally diffused in the cytoplasm and nucleus but translocates to leaky lysosomes rapidly to form visible puncta when LMP takes place (25). We found that at as early as 1 h after treatment with quinacrine, mCherry-galectin-3 puncta were visualized (Figure 4C). Quinacrine is a fluoresced chemical, which is known to be intercalative to DNA and lysosomotropic to lysosome as shown in the left panel of Figure 4C, colocalized with gelactin-3 puncta in lysosomes (right panel). Therefore, we hypothesized that quinacrine blocks lysosomal degradation of DR5, and possibly other components of DISC, likely by inducing LMP. Lysosomal degradation of death receptors and its role in extrinsic apoptotic pathways require further investigation.

### Anti-tumor Effect of Quinacrine in Combination With TRIAL in Ovarian Cancer Xenograft Model

**Figure 5:**
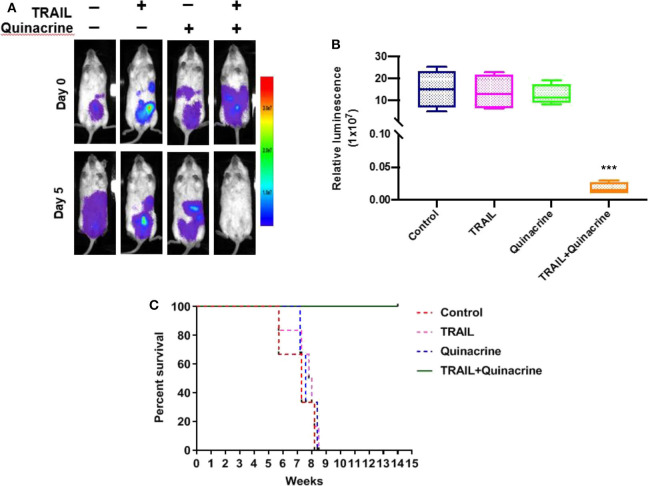
Quinacrine overcome TRAIL resistance *in vivo*. A2780-luc cells were injected into the peritoneum of NSG mice, 5 × 10^6^ cells/mouse, on day −5. Five days after inoculation, mice were imaged on day 0 using the Lago X system. Mice were treated with intraperitoneal injection of quinacrine at 50 mg/kg on day 1 and TRAIL at 10 mg/kg on day 1, 2, 3, and 4. On day 5, mice were reimaged. **(A)** Bioluminescent images of one representative mouse of each group before and after treatment. **(B)** Bioluminescence signal of intraperitoneal tumors treated for 4 weeks compared with pretreated tumors (data are means ± S.D.; *n* = 5, ***p < 0.001). **(C)** Kaplan-Meier survival of mice with different treatment (*n* = 5/group).

We chose ovarian cancer for our *in vivo* study because of its unique feature of local spreading within abdomen and pelvis and practical intraperitoneal delivery of chemotherapeutics that has been proved more effective than systemic delivery under certain circumstances. After treatment, we detected minimum bioluminescence in the group of mice treated with quinacrine and TRAIL, while mice treated with quinacrine or TRAIL alone showed no difference compared to the vehicle control group (Figure 5A, one representative mouse from each group; Figure 5B, bioluminescent intensity captured on day 5). Treatment was repeated with an additional cycle to each group and then subjected to observation. Mice in control group, and groups of quinacrine alone and TRAIL alone died in about 8 weeks (Figure 5C), however, mice treated with combination of TRAIL and quinacrine survived for 20 weeks without tumor growth when they were euthanized.

## Discussion

Ovarian cancer (epithelial ovarian, fallopian tube, or primary peritoneal cancer) (26) is the fifth most common cause of cancer-related death of women in the US with an estimated 22,530 new cases and 13,980 deaths in 2019 (American Cancer Society. Cancer Facts & Figures 2019). Due to lack of an effective screening test (27), ovarian cancer typically presents at a later stage (Stage III or IV) and one third of patients have malignant ascites at initial presentation (28). Current treatment of ovarian cancer is primarily limited to surgery and chemotherapy (NCCN guidelines). Five-year survival is ~47.6% in the United States (SEER Cancer Statistics Review). Thus, new treatments are urgently needed to help patients suffering from this deadly disease.

Quinacrine, introduced in the 1920's, was originally used for malaria treatment and prophylaxis (29, 30), treatment of giardiasis (31), lupus (32), rheumatoid arthritis (33), and for female sterilization (34). Quinacrine was initially developed as an oral drug absorbed in the gastrointestinal track that accumulates in liver with a concentration 20,000 times higher than that of plasma (35). An injectable formulation of quinacrine was developed in the United States in 1964, which was approved for treatment of ascites associated with various cancers (36, 37); but drug marketing was discontinued in 1977, and the NDA (New Drug Application) was withdrawn in 2003 (38, 39).

Most recently, quinacrine emerged as a potential anti-cancer agent. Quinacrine is actively involved in multiple signaling pathways including apoptosis, cell cycle arrest, DNA repair, autophagy, and arachidonic acid metabolism (29, 30) by targeting p53 (17, 40), DR5 (16, 41), MCL-1 (16, 42), NFkB (40), and others in cancer cells. The anti-cancer effects of quinacrine have been tested in various preclinical cancer models (43–46). Quinacrine by itself administered either by oral gavage or intraperitoneal delivery has shown limited anticancer effects in animal models. It shows more or less synergistic effective in combination with other anticancer agents in tumor suppression (42) or survival prolongation (47). Clinical trials using quinacrine as a single agent or in combination with other therapeutics are ongoing in various cancers [colorectal cancer [NCT01844076], prostate cancer [NCT00417274], non-small cell lung cancer [NCT01839955], renal cancer [NCT00574483], and thyroid cancer (42)]. Overall, quinacrine is well-tolerated with well-established pharmacokinetics and pharmacodynamics in human subjects (48, 49). The question remains how we can step forward to make use of this multi-target, bioactive agent for cancer treatment.

Based on our previous findings that quinacrine can significantly induce DR5 protein levels and synergize with TRAIL to induce apoptosis (16), we combined quinacrine with TRAIL and investigated the antitumor activity of this drug combination in ovarian cancer models. We confirmed synergism between TRAIL and quinacrine in all ovarian cancer cell lines tested, including TRAIL-sensitive (SK-OV-3 and OVCAR-4) and TRAIL resistant (A2780 and OVCAR-8) cells. We further investigated the mechanism of quinacrine-induced DR5 accumulation and found that not only can quinacrine induce significant high levels of DR5 mRNA but can also block DR5 degradation evidenced by significantly prolonged DR5 half-life in quinacrine-treated cells. From a mechanistic standpoint, quinacrine is therefore superior to other DR5 inducers that enhance DR5 mRNA (DNA-damaging agents activate DR5 transcription dependent of p53 (50, 51) or reduce DR5 degradation [by proteasomal inhibitors (52)]. We also found that quinacrine induces DR5 subcellular localization changes and accumulates in lysosomes—an observation that warrants further investigation of the metabolism of death receptor and death signaling. Our preliminary data show that quinacrine induces LMP (lysosomal membrane permeabilization) as indicated by aggregation of galectin-3.

The anti-cancer effects of quinacrine in combination with TRAIL in ovarian cancer xenograft models we observed are extraordinary. Two cycles of one dose of quinacrine (50 mg/kg) followed by four doses of TRAIL (10 mg/kg) resulted in mice that were cancer free for 20 weeks when euthanized while all other mice (vehicle control, quinacrine alone, and TRAIL alone) died ~8 weeks after cancer cell inoculation. No therapeutic effect of TRAIL or quinacrine alone was observed at the doses delivered in our experiments. The remarkable synergistic effect we observed may be partly explained by the route of administration—intraperitoneal injection of quinacrine and TRAIL, instead of oral delivery and systemic injection, respectively, which we hypothesize may lead to longer exposure of cancer cells to therapeutic agents in higher concentrations within the peritoneum.

Intraperitoneal delivery of therapeutics in the adjuvant setting is a practical approach and has been exclusively practiced as an effective way to treat ovarian cancer allowing close contact of tumor cells with therapeutic agents at higher concentrations in the peritoneal cavity, which is the principle site of spread and recurrence of ovarian cancer (53, 54). It has been shown that intravenous paclitaxel and intraperitoneal cisplatin or carboplatin on day 1, and intraperitoneal paclitaxel on day 8 every 21 days for 6 cycles for stage II, III, or IV ovarian cancer patients who obtained optimal surgical debulking showed significant prolonged overall survival (55). In our experiments, intraperitoneal delivery of quinacrine and TRAIL showed significant therapeutic efficacy to eliminate cancer cells in the peritoneal cavity.

In summary, we show that intraperitoneal delivery of quinacrine and TRAIL is rational and practical with remarkable synergistic anti-cancer effects in preclinical models of ovarian cancer. Clinical investigation of combining quinacrine TRAIL for ovarian cancer treatment is warranted.

## Data Availability Statement

All datasets generated for this study are included in the article/Supplementary Material.

## Ethics Statement

This animal study was reviewed and approved by City of Hope IACUC.

## Author Contributions

RL: design and carry out most of the laboratory work and drafted the manuscript. YY: LMP assay and animal work. GW: TRAIL purification and animal work. EY: revise work of figures and manuscript. GY: animal work. XQ and LZ: tissue culture and cytotoxicity assay. YW: TRAIL expression vector construction and purification. TZ: design of the work and critical input in interpretation of the work. YZ: critical input and analysis and interpretation of data for the work. EW: substantial contributions to the conception and design of the work, analysis and interpretation of data, draft, revise, and finalize the manuscript. All authors contributed to the article and approved the submitted version.

## Conflict of Interest

The authors declare that the research was conducted in the absence of any commercial or financial relationships that could be construed as a potential conflict of interest.

## Supplementary Material

Supplementary Figure 1 Combination index of TRAIL and quinacrine in SK-OV-3, OVCAR-4, OVCAR-8, and A2780 cell lines as calculated by CompuSyn software. Synergy, additivity, and antagonism are defined as CI < 1, CI = 1, and CI > 1, respectively.
