# Supplementary material for: Repositioning Quinacrine Toward Treatment of Ovarian Cancer by Rational Combination With TRAIL
Source: Front Oncol. 2020 Jul 16;10:1118. doi: 10.3389/fonc.2020.01118 (PMC7379129; doi:10.3389/fonc.2020.01118)
Supplement: Supplementary Figure 1 — Combination index of TRAIL and quinacrine in SK-OV-3, OVCAR-4, OVCAR-8, and A2780 cell lines as calculated by CompuSyn software. Synergy, additivity, and antagonism are defined as CI < 1, CI = 1, and CI > 1, respectively. [file Data_Sheet_1.docx]

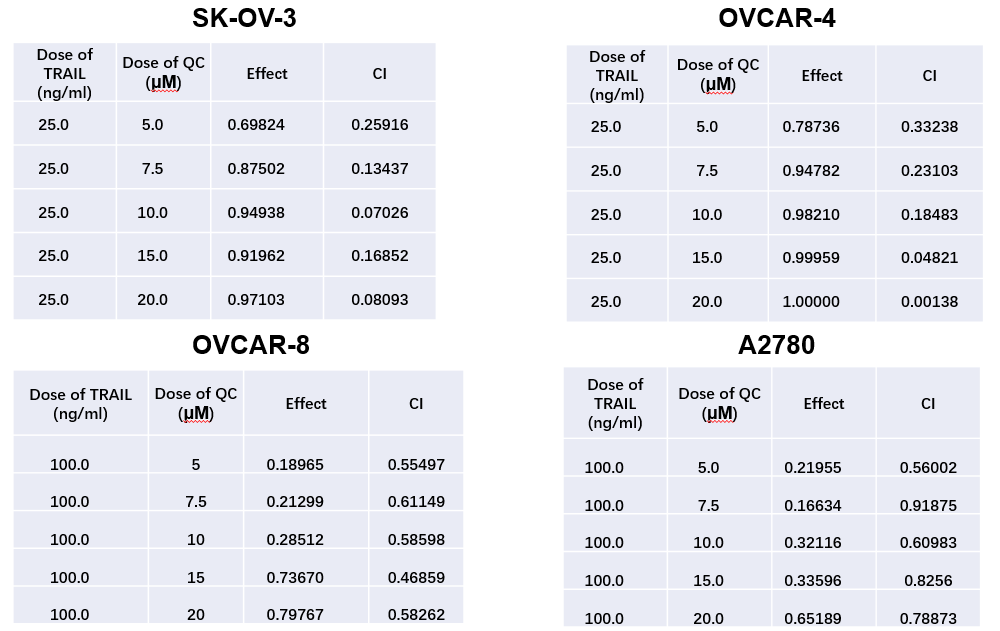


**Supplementary figure 1.** Combination index of TRAIL and quinacrine in SK-OV-3, OVCAR-4, OVCAR-8 and A2780 cell lines as calculated by CompuSyn software. Synergy, additivity, and antagonism are defined as CI < 1, CI = 1, and CI > 1, respectively.
